# Preliminary assessment of neuropathy progression in patients with hereditary ATTR amyloidosis after orthotopic liver transplantation (OLT)

**DOI:** 10.1186/1750-1172-10-S1-P19

**Published:** 2015-11-02

**Authors:** David Adams, Juan Buades, Ole Suhr, Laura Obici, Teresa Coelho

**Affiliations:** 1 Centre Paris-Sud, APHP, Hopital de Bicetre and Centre de Reference National des Neuropathies Amyloides Familiales (NNERF), Service de Neurologie, 94275, LE KREMLIN-BICETRE, France; 2 Hospital Son Llatzer, Servicio de Medicina Interna, 7198, Palma de Mallorca, Spain; 3 Umea University, Department of Public Health and Clinical Medicine, SE-90185, Umea, Sweden; 4 Fondazione IRCSS Policlinico,) Amyloid Research and Treatment Centre, 27100, Pavia, Italy; 5 Hospital de Santo Antonio, Unidade Clinica de Paramiloidose, 4099-001, Porto, Portugal

## Background and objectives

Familial amyloidotic polyneuropathy (FAP) is a fatal, autosomal dominant disease caused by abnormal tissue deposition of mutant and wild-type transthyretin (TTR) in peripheral nerves, gastrointestinal tract, and heart. Since the liver is the primary source of WT and mutant TTR, orthotopic liver transplantation (OLT) has been the standard of care in select patients. Despite undergoing OLT, some patients experience progression of their underlying disease. There is evidence to suggest that disease progression after OLT is caused by ongoing deposition of WT TTR protein produced by the transplanted liver. In order to better understand the natural history of patients receiving FAP-OLT, we performed a retrospective assessment of time to polyneuropathy disability (PND) score progression.

## Methods

We characterized neuropathy severity with the (PND) score. Descriptive statistics including proportions of FAP patients who progress by at least one PND stage after OLT and median time to PND stage progression were assessed in available FAP-OLT patients in Sweden, Portugal, Spain, Italy and France from 1991-2015. The Italian cohort only included patients that had progressed, while patients from other countries included all-comers (progressors and non-progressors).

## Results

A total of 362 FAP-OLT patients were preliminarily assessed from: Portugal, 67; Spain, 59; Sweden, 29; France, 200; Italy, 7. Patients from Portugal, Spain, and Sweden all had the Val30Met (V30M) TTR variant, whereas 69% and 14% of patients from France and Italy had the V30M TTR variant, respectively. Among all-comers with a pre-OLT PND score (n=322/355), 61% underwent transplantation at PND stage of 0 or I, 26% at stage II, 12% at stage IIIa or IIIb, and <1% at stage IV. Within the Italian cohort, 43% underwent transplantation at stage I, 43% at stage II and 14% at stage IIIb. Following OLT, 33% of all-comers (n=107/322) experienced a PND progression; the percent of patients who progressed varied by country (Sweden, 31%; Portugal, 21%; Spain, 37%; France, 37%). Overall, median time from transplantation to PND progression, was 2.9 years (range: 2.5-4.5 years); median time to progression within the individual countries included: Portugal, 4.5 yrs; Spain, 2.6 yrs; Sweden, 4.0 yrs; France, 2.5 yrs; Italy, 3.0 yrs.

## Conclusions

These preliminary data suggest that despite receiving an OLT, disease progression may not be halted in some patients with FAP. Based on this multi-national population of FAP-OLT patients with both V30M and non-V30M TTR genotypes, clinically meaningful neuropathy progression was observed in a median of 2.9 years after OLT. These findings suggest the need to carefully monitor FAP-OLT patients for signs of progression.

